# Deep learning assisting the surgical management of gynecologic cancers

**DOI:** 10.1097/CCO.0000000000001254

**Published:** 2026-06-22

**Authors:** Veronica Tius, Martina Arcieri, Stefano Restaino, Giuseppe Vizzielli

**Affiliations:** a Clinic of Obstetrics and Gynecology, “S. Maria della Misericordia” University Hospital, Azienda Sanitaria Universitaria Friuli Centrale (ASUFC); b Medical Area Department (DAME), in Department of Medicine (DMED), University of Udine, Udine, Italy

**Keywords:** artificial intelligence, deep learning, gynecology, oncology, surgery

## Abstract

**Purpose of review:**

This review examines the current role of deep learning in the surgical management of gynecologic cancers. It aims to evaluate applications across surgical training, intraoperative guidance, and outcome prediction, while identifying limitations that hinder translation into routine clinical practice.

**Recent findings:**

Deep learning-based systems show promising performance in enhancing surgical skills through objective assessment and simulation platforms. Intraoperatively, deep learning models can recognize anatomical structures, surgical phases, and tumor tissue, with high diagnostic accuracy in selected settings. Emerging techniques such as surgical optomics and hyperspectral imaging may further improve tumor detection. Additionally, predictive models integrating clinical, imaging, and intraoperative data demonstrate potential in estimating complications, resource utilization, and survival outcomes. However, most studies are retrospective, based on limited datasets, and lack external validation, with minimal evidence of real-world clinical impact.

**Summary:**

Artificial intelligence, particularly deep learning algorithms, represents a rapidly evolving tool in gynecologic oncologic surgery, with potential to standardize and personalize care. Nevertheless, significant gaps remain between experimental performance and clinical implementation.

## INTRODUCTION

Surgical management of gynecologic cancers remains one of the most complex areas in oncologic care, requiring the integration of technical skill, anatomical understanding, real-time decision-making, and accurate interpretation of tumor biology. Variability in surgical performance, intraoperative judgment, and patient selection continues to represent a major challenge, directly impacting oncologic outcomes and perioperative morbidity.

Artificial intelligence (AI), particularly deep learning (DL), has emerged as a promising tool to address these limitations by enabling the analysis of high-dimensional data such as medical images, surgical videos, and clinical variables. DL-based models have shown increasing capabilities in pattern recognition and predictive modeling, suggesting applications across the entire surgical pathway – from training and preoperative planning to intraoperative guidance and postoperative outcome prediction.

Within gynecologic oncology, AI integration into surgical practice is still in its early stages but is rapidly evolving [1^▪^]. Current research focuses on three main domains: enhancement of surgical training, intraoperative assistance through real-time recognition of anatomical structures and tumor tissue, and prediction of surgical and oncologic outcomes through multimodal data integration. These developments may support a more standardized, data-driven surgical approach, potentially reducing variability and improving patient-specific decision-making.

However, despite the growing body of evidence and promising performance metrics, substantial gaps remain between experimental development and clinical implementation. Most models rely on limited datasets, lack external validation, and are not yet integrated into routine workflows. In addition, issues related to interpretability, reproducibility, and ethical responsibility continue to limit their adoption in real-world clinical settings.

This review explores the current applications of deep learning in the surgical management of gynecologic cancers, critically evaluating their potential, limitations, and future role in clinical practice. 

**Box 1:**
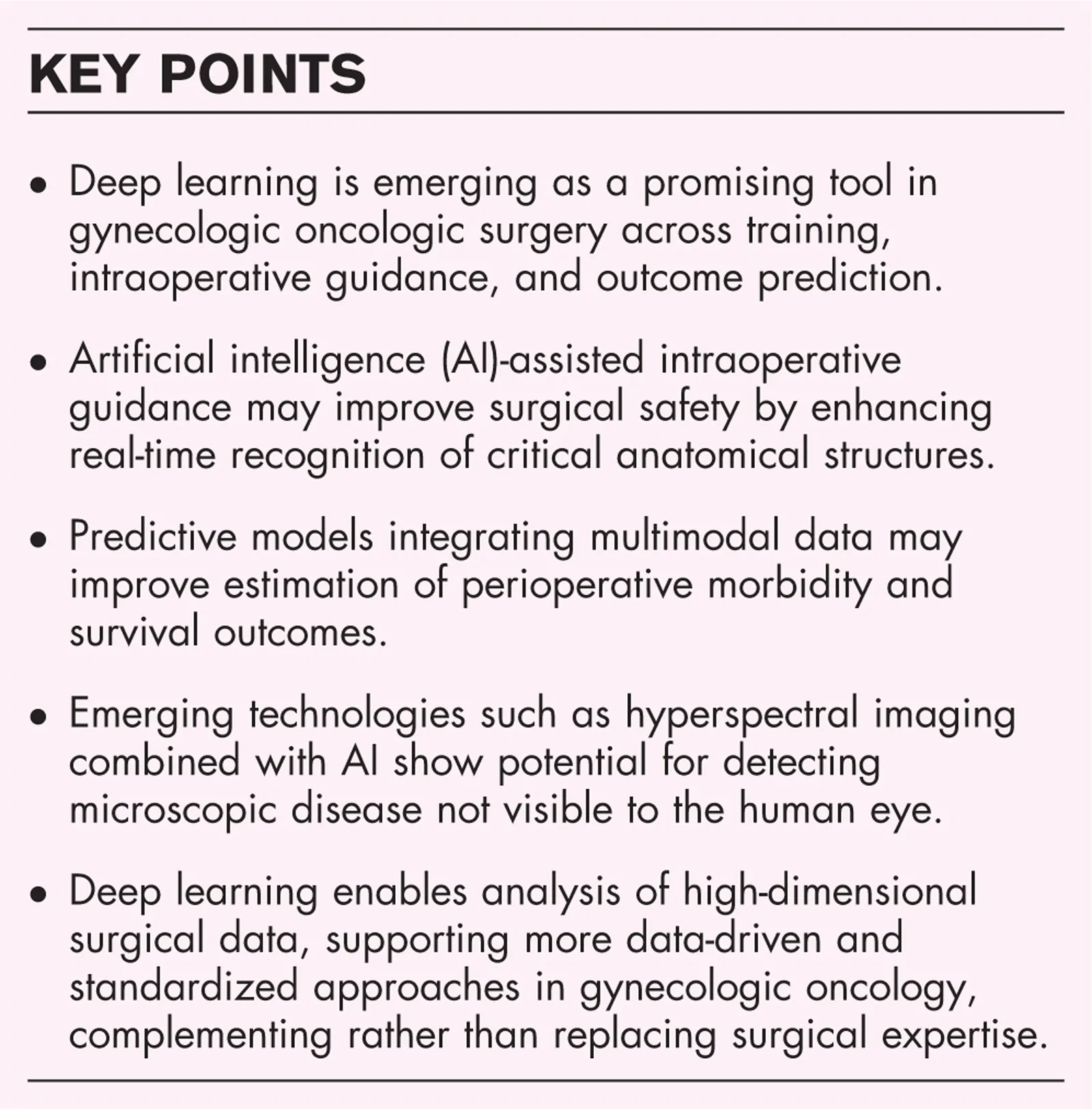
no caption available

## ARTIFICIAL INTELLIGENCE IN SURGICAL MANAGEMENT OF GYNECOLOGICAL CANCERS: ENHANCING SURGICAL SKILLS ACQUISITION

The use of deep learning in gynecologic oncologic surgery begins at the level of surgical training and skill development. Traditional training models rely on progressive exposure and mentorship-based learning. Although effective, this approach is inherently limited by variability in case volume, institutional expertise, and subjective assessment of surgical performance.

AI-integrated platforms, including pelvic trainers, virtual simulators, and immersive reality environments, offer the potential for more standardized and objective surgical training [2^▪▪^]. Deep learning models have demonstrated high accuracy in evaluating complex surgical tasks, providing automated performance metrics and feedback [3^▪^,^4^^▪▪^,5^▪^]. These systems can decompose procedures into discrete steps, enabling targeted feedback and potentially accelerating skill acquisition.

However, despite these advances, most AI-based training tools remain confined to experimental settings and lack widespread clinical validation. Many models are trained on limited datasets and do not adequately capture variability in surgical techniques or patient anatomy. Moreover, the extent to which AI-driven training translates into improved real-world surgical outcomes remains unclear.

Thus, while AI has the potential to standardize surgical education, its impact on clinical performance and patient safety has yet to be clearly established.

## ARTIFICIAL INTELLIGENCE IN SURGICAL MANAGEMENT OF GYNECOLOGICAL CANCERS: PROVIDING INTRAOPERATIVE GUIDANCE

Artificial intelligence has demonstrated a rapidly expanding role in gynecologic oncologic surgery, particularly through the integration of imaging, clinical, surgical, and biological data to support intraoperative decision-making. Deep learning models applied to laparoscopic images and videos have shown promising potential for recognizing surgical phases and instruments, detecting cancer, predicting surgical morbidity, and estimating complete cytoreduction and survival outcomes. However, most applications remain at a developmental stage and are not yet routinely integrated into clinical practice, as current evidence is largely based on retrospective datasets and proof-of-concept studies.

### Anatomical and instrument recognition

AI-based systems, particularly deep learning models, have the potential to assist surgeons in real time by improving recognition of surgical phases, instruments, and key anatomical landmarks, including ureters and major vessels [6^▪^,^7^^▪▪^]. These capabilities are especially relevant in minimally invasive gynecologic oncology, where limited tactile feedback and reliance on visual cues increase procedural complexity.

Real-time identification of anatomical structures may enhance intraoperative safety by reducing the risk of inadvertent injury and improving surgical orientation. In addition, automated instrument detection and workflow segmentation may support more standardized surgical execution.

However, most models are trained on curated datasets and may not generalize well to real surgical variability. Differences in anatomy, technique, and intraoperative conditions – such as bleeding, smoke, and tissue distortion – can significantly affect performance. Moreover, prospective evidence demonstrating a direct impact on surgical outcomes is still lacking, limiting clinical integration.

### Cancer detection

Among the most promising applications of artificial intelligence in gynecologic oncologic surgery is intraoperative cancer detection. Deep learning models have already shown high diagnostic performance in procedures such as hysteroscopy. Wang *et al.*[8^▪▪^] reported a system achieving an area under the curve (AUC) of 0.979 and an accuracy of 94.1% in detecting atypical endometrial hyperplasia and endometrial cancer. These approaches may improve diagnostic accuracy, reduce missed lesions, and support surgical planning, although their real-time intraoperative applicability remains limited.

Increasing attention has been given to surgical optomics, defined as the integration of optical imaging technologies with computational analysis to provide functional information during surgery [9^▪^]. Hyperspectral and multispectral imaging appear particularly promising, enabling tissue characterization based on molecular and metabolic signatures beyond the visible spectrum.

Recent studies have explored the use of deep learning combined with spectroscopic imaging for tissue segmentation and tumor classification in gynecologic cancers [10^▪▪^,^11^^▪▪^,12^▪^]. These technologies may allow detection of microscopic disease, including peritoneal carcinomatosis and nodal micrometastases, often not visible with conventional imaging. In this context, it is possible to envision a role for surgical optical biopsy applied to laparoscopic instruments, enabling real-time image guidance during surgery. Laparoscopic cameras integrated with deep learning models and miniaturized spectral sensors could be developed for direct intraoperative application, allowing the extraction of quantifiable tissue features during surgery, as suggested by previous studies [13^▪▪^].

Nevertheless, most of these approaches remain experimental. Technical challenges – including hardware integration, processing speed, and real-time interpretation – represent major barriers to clinical implementation. Importantly, no system has yet demonstrated a clear improvement in intraoperative decision-making or oncologic outcomes, highlighting the gap between technological development and clinical utility.

### Complication following surgery

Several deep learning–based tools have been proposed to predict perioperative and postoperative outcomes in gynecologic oncology surgery. Artificial intelligence offers the potential to anticipate surgical risk and support perioperative management through the integration of demographic, clinical, and intraoperative variables.

The Leeds L-AI-Ovarian Surgery score [14^▪^] represents one of the first applications of this approach, aiming to estimate length of hospital stay following cytoreductive surgery. By combining demographic and intraoperative data, this model demonstrated the feasibility of AI in predicting resource utilization and postoperative recovery, potentially supporting patient counseling and healthcare planning. Beyond short-term outcomes, deep learning has also been applied to predict treatment-related complications affecting long-term quality of life. For example, Han *et al.*[15^▪^] developed a model predicting vaginal stenosis in cervical cancer patients undergoing postoperative radiotherapy, achieving an AUC of 0.95. Such tools may enable early identification of high-risk patients and facilitate preventive strategies and tailored follow-up.

More broadly, these models highlight the potential of artificial intelligence to shift perioperative care from reactive to proactive care, thereby improving the personalization of treatment pathways. In gynecologic oncology, where surgical morbidity significantly impacts both oncologic outcomes and quality of life, predictive tools may contribute to optimized patient management.

However, current evidence remains limited and largely exploratory. Most models are based on retrospective analyses and lack external validation, raising concerns about reliability and generalizability. In addition, their integration into clinical workflows is still limited, as predictive outputs are not incorporated into standardized decision-making pathways. The absence of prospective studies demonstrating improved outcomes represents a major barrier to clinical adoption.

Finally, many models focus on single outcomes and fail to capture the complexity of perioperative risk, which is influenced by multiple interacting factors, including surgical technique, tumor biology, and patient frailty. Future research should focus on integrated models combining clinical, surgical, and biological data, validated across multicenter datasets and implemented in real-world settings.

### Survival outcomes prediction

Artificial intelligence has increasingly been explored for predicting oncologic outcomes in gynecologic cancers, particularly in the context of surgical decision-making. Deep learning and machine learning models can integrate clinical, radiological, and intraoperative data to estimate survival and treatment response, potentially enabling more personalized therapeutic strategies.

In ovarian cancer, the ANAFI score proposed by Laios *et al.*[16^▪^] represents one of the first applications of machine learning to predict the likelihood of complete cytoreduction and survival outcomes based on intraoperative tumor distribution. This approach emphasizes the role of spatial tumor burden as a key determinant of surgical success and prognosis.

More recently, deep learning models have been applied to laparoscopic imaging to predict progression-free survival. Ma *et al.*[17^▪▪^] developed a model capable of distinguishing between short and long progression-free survival using tumor morphology assessed during diagnostic laparoscopy, achieving an AUC of 0.819. These findings support the concept that tumor phenotype, as captured by imaging, may reflect underlying tumor biology and influence clinical outcomes.

In cervical cancer, predictive models combining imaging and clinicopathological variables have shown high performance in estimating survival outcomes. The DeepSurv algorithm demonstrated an AUC of 0.900 for 5-year overall survival prediction [18^▪^], while convolutional neural network–based models integrating MRI data achieved AUC values above 0.88 for recurrence-free survival prediction [19^▪^]. These approaches may support treatment selection, particularly when the choice between surgery and chemoradiotherapy is uncertain, and may assist in tailoring surgical radicality.

However, despite promising performance metrics, the clinical applicability of these models remains limited. Most studies are retrospective, often single-center, and lack external validation, limiting generalizability. Moreover, high predictive accuracy does not necessarily translate into clinical utility, as integration into surgical decision-making workflows is not yet standardized and evidence of improved patient outcomes is lacking.

Another major limitation is the limited interpretability of many deep learning models, which may hinder clinical acceptance. Understanding how predictions are generated is essential in high-stakes oncologic decisions, where transparency and trust are critical.

Future research should focus on integrating imaging, clinical, and molecular data to improve prognostic stratification and support truly personalized surgical oncology.

## ARTIFICIAL INTELLIGENCE IN SURGICAL MANAGEMENT OF GYNECOLOGICAL CANCERS: MODELING A NEW SURGEON-PATIENT RELATIONSHIP

In parallel with its applications in surgical workflows, artificial intelligence is progressively reshaping the relationship between patients and healthcare professionals. Patients with gynecologic cancers increasingly rely on digital tools and AI-powered platforms to access information regarding diagnosis, surgical risks, treatment options, and survival outcomes [20^▪▪^].

While these tools improve access to information, they also introduce significant risks. AI-generated responses may be inaccurate or misleading, particularly in complex oncologic contexts, due to the phenomenon of “hallucinations.” In such settings, misinformation may undermine patient trust, increase anxiety, and negatively affect shared decision-making.

From this perspective, artificial intelligence should not replace clinical communication but rather function as a complementary tool requiring professional supervision. The physician's role becomes even more central, acting as an interpreter and validator of information, ensuring that AI-generated content is aligned with evidence-based practice.

Initiatives such as ESGO-supported digital platforms illustrate the potential of combining AI capabilities with professional oversight to improve patient education [21^▪^]. However, integration of AI into the surgeon–patient relationship also raises ethical concerns, including accountability for misinformation and the risk of over-reliance on automated systems.

Ultimately, AI is likely to redefine rather than replace the surgeon–patient relationship, shifting the clinician's role toward that of an expert guide within an increasingly data-driven environment.

## DISCUSSION

Artificial intelligence is rapidly emerging as a transformative tool in gynecologic oncologic surgery, with potential applications across training, intraoperative guidance, and outcome prediction. Despite impressive technical performance, a critical gap persists between technological development and clinical implementation. Most studies report high accuracy in controlled environments; however, these results should be interpreted cautiously. Performance in curated datasets does not necessarily translate into real-world clinical utility. Many models rely on small, single-center datasets and lack external validation, limiting generalizability.

Data quality and representativeness remain major challenges. Surgical datasets are inherently heterogeneous and influenced by multiple factors, including surgeon experience, procedural complexity, and institutional variability. Selection bias and inconsistent annotation further affect model robustness. Another key limitation is interpretability. Many AI systems function as “black boxes,” limiting clinicians’ ability to understand and trust their predictions. In oncologic surgery, where decisions are high-stakes, lack of transparency represents a major barrier to adoption.

The heterogeneity of reported performance metrics further complicates comparison across studies. Standardized frameworks such as CONSORT-AI, STARD-AI, and PROBAST-AI are essential to improve transparency and reproducibility.

From a clinical perspective, AI currently plays a supportive rather than transformative role. It may enhance pattern recognition and decision-making but cannot replace human judgment. At present, AI should be considered a decision-support tool rather than an autonomous system.

Future developments should focus on integrating multimodal data – including imaging, clinical, and molecular information – to enable more accurate risk stratification and personalized surgical strategies. This will require robust validation, multidisciplinary collaboration, and appropriate regulatory frameworks. Ethical and medico-legal challenges also remain, particularly regarding responsibility and accountability in AI-assisted decision-making. Addressing these issues will be essential for safe clinical implementation.

In summary, while AI holds significant promise, its current impact in gynecologic oncologic surgery remains limited, and further work is required to translate technological advances into clinical benefit.

## CONCLUSION

Artificial intelligence is rapidly transforming gynecologic oncologic surgery, offering opportunities to improve training, intraoperative decision-making, and outcome prediction. However, despite strong performance in experimental settings, its integration into routine clinical practice remains limited.

Currently, AI should be viewed as a tool to augment, rather than replace, surgical decision-making. The complexity of oncologic surgery requires the integration of technical expertise, clinical judgment, and biological understanding – elements that AI cannot yet fully replicate.

Future progress will depend on the development of large, multicenter datasets, standardized validation frameworks, and improved model interpretability, alongside clear ethical and regulatory guidelines. Ultimately, the goal is not to automate surgery but to enhance surgical reasoning – making it more informed, consistent, and tailored to each patient.

## Acknowledgements

*None.*

### Financial support and sponsorship

*None.*

### Conflicts of interest

*There are no conflicts of interest.*

## REFERENCES AND RECOMMENDED READING

Papers of particular interest, published within the annual period of review, have been highlighted as:

▪ of special interest
▪▪ of outstanding interest
